# Intrafraction organ movement in adaptive MR-guided radiotherapy of abdominal lesions – dosimetric impact and how to detect its extent in advance

**DOI:** 10.1186/s13014-024-02466-x

**Published:** 2024-06-25

**Authors:** Carolin Buchele, C. Katharina Renkamp, Sebastian Regnery, Rouven Behnisch, Carolin Rippke, Fabian Schlüter, Philipp Hoegen-Saßmannshausen, Jürgen Debus, Juliane Hörner-Rieber, Markus Alber, Sebastian Klüter

**Affiliations:** 1 Department of Radiation Oncology, Heidelberg University Hospital, Im Neuenheimer Feld 400, 69120 Heidelberg, Germany; 2 Heidelberg Institute of Radiation Oncology (HIRO), National Center for Radiation Oncology (NCRO), Heidelberg, Germany; 3 Medical Faculty, Heidelberg University, Heidelberg, Baden-Württemberg Germany; 4 National Center for Tumor Diseases (NCT), Heidelberg, Germany; 5 Institute of Medical Biometry (IMBI), Heidelberg University, Heidelberg, Germany; 6 Clinical Cooperation Unit Radiation Oncology, German Cancer Research Center (DKFZ), Heidelberg, Germany; 7 Scientific RT GmbH, Munich, Germany

**Keywords:** Stereotactic body Radiotherapy (SBRT), Stereotactic ablative radiotherapy (SABR), Image-guided Radiotherapy (IGRT), MR-guided adaptive radiotherapy, Plan adaptation, Intrafraction organ movement

## Abstract

**Introduction:**

Magnetic resonance guided radiotherapy (MRgRT) allows daily adaptation of treatment plans to compensate for positional changes of target volumes and organs at risk (OARs). However, current adaptation times are relatively long and organ movement occurring during the adaptation process might offset the benefit gained by adaptation. The aim of this study was to evaluate the dosimetric impact of these intrafractional changes. Additionally, a method to predict the extent of organ movement before the first treatment was evaluated in order to have the possibility to compensate for them, for example by adding additional margins to OARs.

**Materials & methods:**

Twenty patients receiving adaptive MRgRT for treatment of abdominal lesions were retrospectively analyzed. Magnetic resonance (MR) images acquired at the start of adaptation and immediately before irradiation were used to calculate adapted and pre-irradiation dose in OARs directly next to the planning target volume. The extent of organ movement was determined on MR images acquired during simulation sessions and adaptive treatments, and their agreement was evaluated. Correlation between the magnitude of organ movement during simulation and the duration of simulation session was analyzed in order to assess whether organ movement might be relevant even if the adaptation process could be accelerated in the future.

**Results:**

A significant increase in dose constraint violations was observed from adapted (6.9%) to pre-irradiation (30.2%) dose distributions. Overall, OAR dose increased significantly by 4.3% due to intrafractional organ movement. Median changes in organ position of 7.5 mm (range 1.5–10.5 mm) were detected within a median time of 17.1 min (range 1.6–28.7 min). Good agreement was found between the range of organ movement during simulation and adaptation (66.8%), especially if simulation sessions were longer and multiple MR images were acquired. No correlation was determined between duration of simulation sessions and magnitude of organ movement.

**Conclusion:**

Intrafractional organ movement can impact dose distributions and lead to violations of OAR tolerance doses, which impairs the benefit of daily on-table plan adaptation. By application of simulation images, the extent of intrafractional organ movement can be predicted, which possibly allows to compensate for them.

## Introduction

Linear accelerators with integrated magnetic resonance imaging (MR-linacs) allow daily adaptation of treatment plans to compensate for interfractional changes like differences in breathing phase or position variations of organs at risk (OAR). By adapting the plan to the anatomy of the day, coverage of gross tumor volume (GTV) and planning target volume (PTV) can be significantly increased compared to irradiation with the non-adapted plan [1–4]. Additionally, dose to OARs can be reduced, in particular if they are in close proximity to the treated lesion. This reduction of OAR dose becomes especially relevant for treatments of abdominal lesions with highly mobile OARs like duodenum or small bowel close to the PTV [4–11].

The benefit of adaptive magnetic resonance (MR)-guided stereotactic body radiotherapy (SBRT) to compensate for interfractional changes has been shown in many studies. However, current adaptation times are relatively long and durations of one hour or more have been reported [10–13]. During this time, major changes in OAR position might occur, which could have a negative impact on the adaptation result. There is only limited research on the impact of these intrafractional changes on OAR dose, though. Tyagi et al. have shown that intrafractional organ motion could lead to violation of OAR dose-volume constraints in some fractions in treatments of locally advanced pancreatic cancer with abdominal compression [13]. Similar results have been reported by Teoh et al. with violation of dose constraints in about one-third of fractions, also focusing on treatments of pancreatic cancer [14]. Compared to conventionally fractionated treatments, these violations of OAR dose constraints become more relevant in hypofractionated treatment schemes since dose violations in only few fractions have a higher impact. As a consequence, it might become necessary to detect intrafractional shifts in OAR position in advance and possibly compensate for them by creating planning organ at risk volumes (PRVs). The need to use PRVs to account for position variations of OARs during treatment has been described in previous reports and various methods to generate PRVs for gastrointestinal OARs have been developed [15–17].

Large intrafractional OAR movement might reduce or even negate the benefit of daily plan adaptation, but so far only a few studies have investigated the effect of intrafractional changes on OAR dose and it is still unclear whether intrafractional changes need to be compensated in MR-guided radiotherapy. Therefore, the purpose of this study was to evaluate the effect of intrafractional anatomical changes on OAR dose for various abdominal lesions treated with adaptive MR-guided radiotherapy. Furthermore, a method to predict the extent of OAR movement during the adaptation process was evaluated, which is based on information acquired during simulation imaging.

## Materials and methods

### Patient selection

Twenty patients having received online adaptive MR-guided SBRT between February 2020 and June 2021 were included in the analysis. Patient selection was based on the distance between OAR and PTV. Only patients with an overlap between OARs and PTV in at least one adapted fraction or the baseline plan were included in the analysis. Furthermore, analysis focused on OARs overlapping with the PTV, since OAR movement and subsequent changes to OAR dose were expected to be more relevant in these cases. Overall, 151 adapted fractions were evaluated. Four of the selected patients had two or more OARs overlapping with the PTV, leading to a total of 189 observations available for analysis. Evaluation focused on near-point maximum dose for each OAR, which was defined as the dose to 0.5 cc of the volume (D_0.5cc_). A summary of treatment characteristics can be found in Table 1.

**Table 1:** Treatment characteristics

Patient No.	Treatment localization	OAR	No. of fractions	Prescribed dose (Gy)	OAR constraint D_0.5cc_ (Gy)
1	Liver	Small bowel	10	50	43.5
2	Liver	Stomach	5	50	35
		Duodenum			35
		Esophagus			34
3	Liver	Small bowel	10	50	43.5
4	Liver	Small bowel	10	50	43.5
5	Liver	Duodenum	10	50	43.5
6	Adrenal gland	Small bowel	5	50	35
7	Liver	Small bowel	5	50	35
8	Liver	Duodenum	5	50	35
9	Liver	Stomach	5	50	35
10	Liver	Small bowel	10	50	43.5
		Duodenum			43.5
11	Lymph node	Stomach	6	30	37
12	Adrenal gland	Stomach	5	50	35
13	Adrenal gland	Stomach	8	40	40
		Small bowel			40
14	Abdominal lymph node	Small bowel	7	35	37
15	Liver	Small bowel	10	50	43.5
16	Liver	Duodenum	10	50	43.5
17	Adrenal gland	Small bowel	10	50	43.5
		Stomach			42.5
18	Adrenal gland	Duodenum	5	50	35
19	Pancreas	Small bowel	5	50	35
20	Liver	Small bowel	10	50	43

Overview over treatment localization, OAR used for evaluation, number of treatment fractions, prescribed dose and tolerance dose of the OAR for the respective prescription. For all patients, dose distribution inside the PTV is inhomogeneous with an allowed maximum dose of 125% of the prescribed dose

### Treatment planning

Patient treatment was performed on a 0.35T MR-linac (MRIdian Linac®, ViewRay Inc., Oakwood, USA). Simulation and treatment planning workflow have previously been described in detail [18]. In short, one week prior to the start of patient treatment a 3D MR image (in-plane resolution: 1.5 × 1.5 mm², slice thickness: 3 mm) was acquired in inspiration breath-hold for treatment planning (MRI_Sim_). For most patients, image acquisition was repeated multiple times during the simulation session in order to test robustness of patient position and breath-hold. OARs and GTVs were contoured on one of the available MRI_Sim_. A CTV was then created by adding a 2 mm to 5 mm margin to the GTV, depending on tumor type and location. Subsequently, the CTV was expanded by 3 mm to form the PTV. The prescribed dose should cover at least 95% of the PTV, tolerating inhomogeneities inside the PTV of up to 125% of the prescribed dose. In some cases, PTV coverage needed to be reduced below 95% since OAR dose constraints were always prioritized.

### Adaptation workflow

**Fig. 1:**
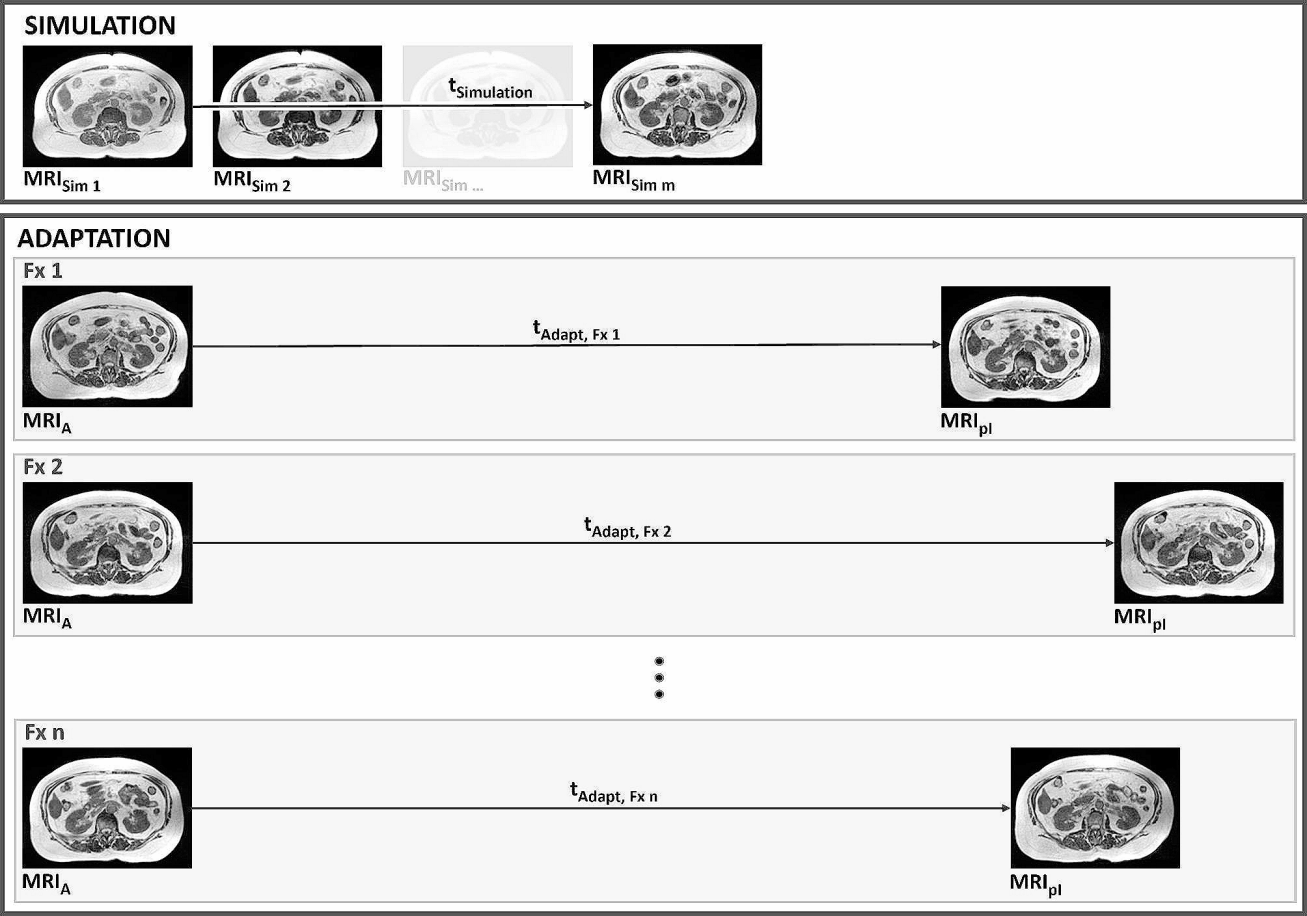
Overview of MR images acquired during simulation and adaptation. At least two MR images were acquired during simulation (MR_Sim 1_ and MR_Sim 2_), but in some cases up to m images were acquired (MR_Sim m_). The simulation time was determined between MR_Sim 1_ and MR_Sim m_. At the beginning of each adapted fraction an MR image was acquired as a basis for the following adaptation workflow (MR_A_). Immediately before the start of irradiation, a second image was acquired (MR_pI_) to check patient position

At the start of each treatment fraction a 3D MR image (MRI_A_) was acquired with the same settings used for MRI_Sim_. MRI_A_ was then registered to MRI_Sim_ using the GTV as a reference structure. Contours were either rigidly (GTV, CTV, PTV) or deformably (OARs) transferred to MRI_A_ and adjusted to the anatomy of the day. Recontouring of organs at risk was performed in a region of 3 cm in medio-lateral and anterior-posterior direction and 1 cm in cranio-caudal direction around the PTV, termed PTV_Expand_ [19]. The baseline plan was re-calculated on MRI_A_ and if either PTV coverage was insufficient or OAR tolerance doses were violated, the plan was re-optimized. After final plan approval from the treating physician, a second pre-irradiation 3D MR image was acquired (MRI_pI_) to check and, if necessary, correct patient position. These verification images were not included in the routine workflow provided by the manufacturer and as a result they were not necessarily acquired at other MR-linac sites on a regular basis. However, their acquisition enabled the evaluation of intrafractional changes to OAR dose as well as analysis of the magnitude of OAR movement during adaptation in this study. An overview of MR images acquired during simulation and adaptation can be found in Fig. 1. Identical to the simulation workflow, image acquisition during adaptation was performed in inspiration breath-hold.

### Dose to organs at risk analysis

Pre-irradiation dose distributions were retrospectively generated by propagating the adapted plan to MRI_pI_. Since MRI_pI_ was acquired immediately before irradiation, this dose distribution was assumed to correspond closely with the irradiated OAR dose. Calculation of pre-irradiation OAR dose also involved retrospective contouring of OARs, in analogy to the online adaptive workflow only within the expanded region around the PTV. D_0.5cc_ values were extracted from adapted (D_0.5cc, A_) and pre-irradiation dose distributions (D_0.5cc, pI_) to evaluate intrafractional changes to OAR dose. To account for different fractionation concepts, these dose values were evaluated relative to OAR tolerance dose (D_0.5cc, TD)_.

### Organs at risk range of movement

In order to analyze OAR movement between MR images, a method was implemented to determine the range of OAR movement during both adaptive treatment (RoM_Adapt_) as well as during treatment simulation (RoM_Sim_). RoM_Sim_ was then compared to RoM_Adapt_ to evaluate the similarity between OAR movement during simulation and adaptation.

In a first step, the contours relevant for analysis of OAR movement were transferred to the same MR image. For each adapted fraction, MRI_pI_ was registered rigidly to MRI_A_ using the GTV contour. The GTV was identical in both images because the contour was rigidly transferred from MRI_A_ to MRI_pI_ during image registration in the adaptive workflow. The OAR contour from MRI_pI_ (OAR_pI_) was then transferred to MRI_A_ so that both contours, OAR_pI_ and the original OAR contour from MRI_A_ (OAR_A_), existed in one image.

A similar workflow was used for simulation images: Since OAR and target volume contours only existed on the one MRI_Sim_ used for treatment planning, the OARs relevant for analysis needed to be contoured on all other MRI_Sim_. Subsequently, MRI_Sim_ images were registered rigidly using the visible tumor structure and all OAR contours from consecutive MR images were transferred to the first simulation image (MRI_Sim 1_) so that all OAR contours were present in one image. Simulation images with large differences in breathing phase or positioning due to patient movement were excluded from the analysis since rigid image registration was not possible in these cases. Image registration and transfer of structures was performed in RayStation® 11B (RaySearch Laboratories AB, Stockholm, Sweden).

**Fig. 2:**
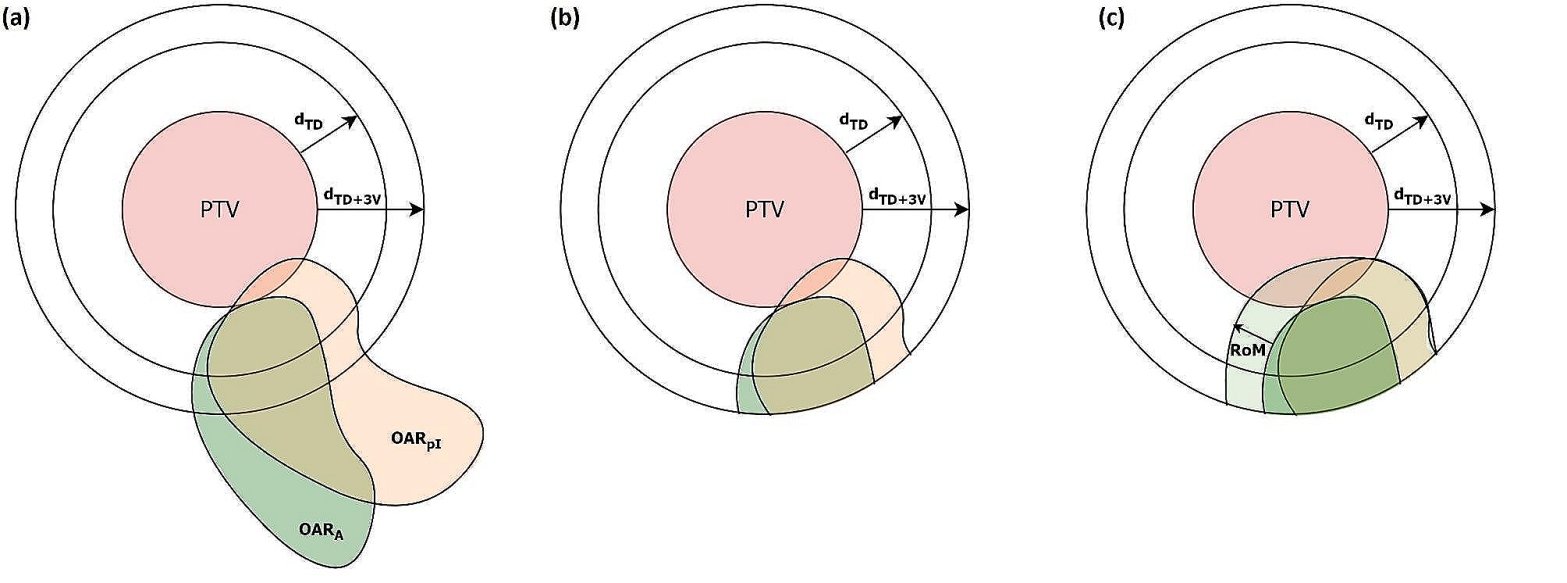
Schematic of distances and structures used for determination of RoM. (**a**) Contours of OAR_A_ and OAR_pI_ are both present in the same MR image. d_TD_ is determined by calculating the mean distance of OAR tolerance isodose to the PTV. d_TD+3V_ is created by adding the in-plane voxel-length times three. (**b**) OAR_A_ and OAR_pI_ are cropped so only parts of the contour inside the area PTV + d_TD+3V_ remain. (**c**) RoM is the margin added to the cropped OAR_A_ contour until it completely surrounds the cropped OAR_pI_ contour

In the next step, an area around the PTV was defined in which the following range of organ movement (RoM) analysis was performed. To determine this area, the mean distance of the OAR tolerance isodose to the PTV (d_TD_) was calculated and an in-plane length of 3 image voxels was added (d_TD+3V_), so that the volume of the subsequently cropped OAR was sufficiently large for analysis (Fig. 2(a)). The mean distance to the tolerance isodose was used because conformity might be low in some cases due to a large dose fall-off in one direction in order to meet the OAR tolerance dose. Then, OARs were cropped so that only the part around the PTV expanded isotropically by d_TD+3V_ remained (Fig. 2(b)). In the last step, an automatic routine was used which continually expanded one OAR contour isotropically by the in-plane length of one voxel until it completely covered the other OAR contour present on the same MR (Fig. 2(c)). In case of the adapted fractions, OAR_A_ was expanded until it covered OAR_pI_. The resulting margin used for expansion was defined as RoM. All steps involved in this evaluation including resulting structures and distances are shown schematically in Fig. 2. The same routine was run on simulation images. The base plan was used to define d_TD_ and the original OAR contour from MRI_Sim1_ was expanded until it covered OAR contours from consecutive simulation images. If more than two simulation images existed the routine was also used on OAR contours from directly successive MRI_Sim_. In this case, multiple RoM_Sim_ were available and the maximum RoM_Sim_ value was selected to determine the largest OAR movement during simulation. This maximum value was then used for comparison with RoM_Adapt_. The automatic routine used for RoM analysis of OARs was implemented in MATLAB R2021a (The MathWorks, Inc., Natick, MA, USA).

Furthermore, the correlation between the duration of simulation sessions and magnitude of organ movement was evaluated. Simulation sessions usually took considerably less time than the majority of current adaptation sessions. Therefore, the aim of this sub-analysis was to indicate whether large organ movements, which could negatively impact the adaptation result, might also happen in a short time frame.

### Statistical analysis

All statistical tests were performed in R version 4.2.2. A linear mixed effects model was fit to analyze the change in OAR dose between adapted and pre-irradiation dose distributions. The dose distribution version was included as fixed effects. To account for the evaluation of multiple OARs in some patients, OAR nested within patients were entered as random intercepts. Additionally, the change in occurrence of tolerance dose violations between adapted and pre-irradiation version was evaluated using McNemar mid-P test.

Pearson correlation was used to evaluate the correlation between simulation time and percentage of fractions where RoM_Adapt_ was equal or smaller than maximum RoM_Sim_. The same method was applied to determine if maximum RoM_Sim_ was dependent on simulation times.

## Results

### Dose to organs at risk analysis

**Fig. 3:**
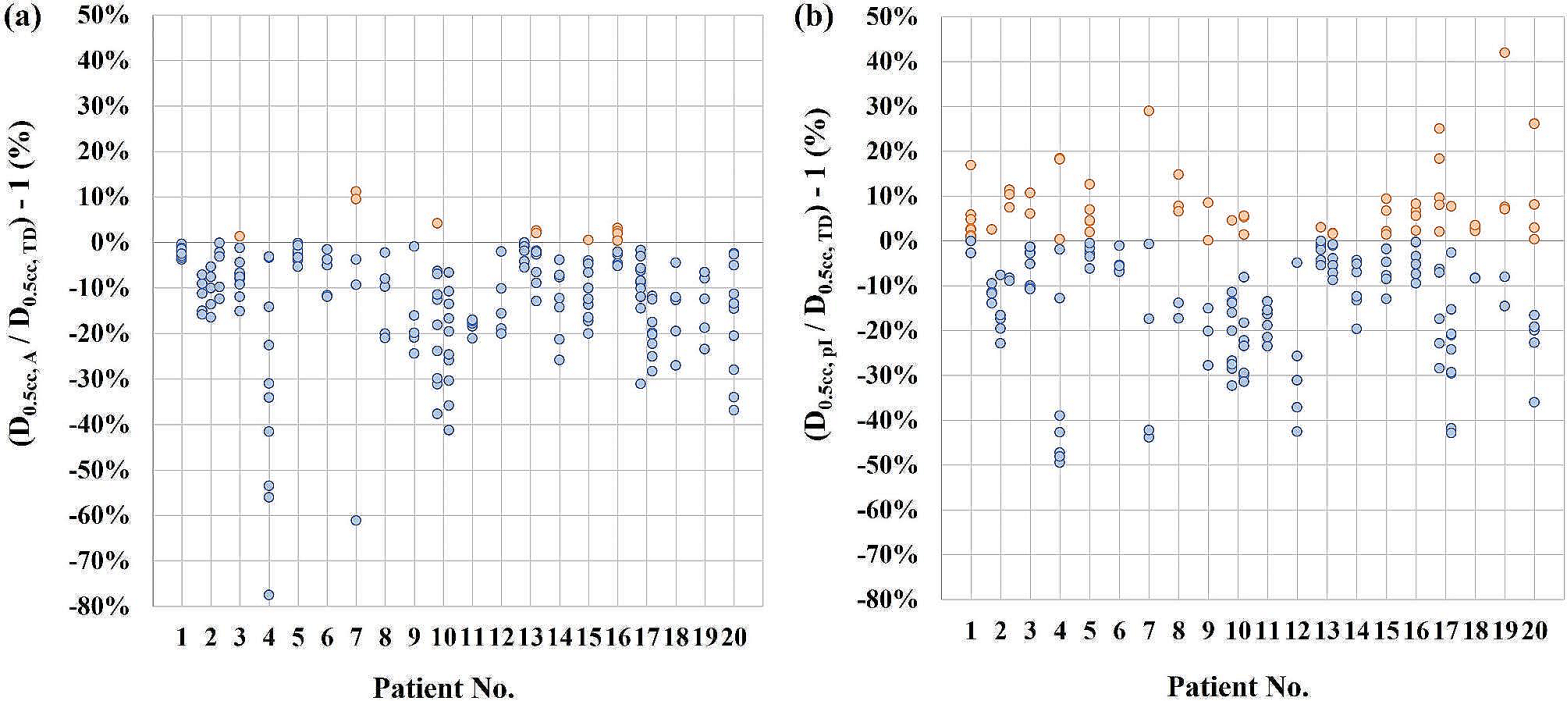
OAR dose relative to the respective dose constraint. (**a**) Adapted dose distribution version. (**b**) Pre-irradiation dose distribution version

**Fig. 4:**
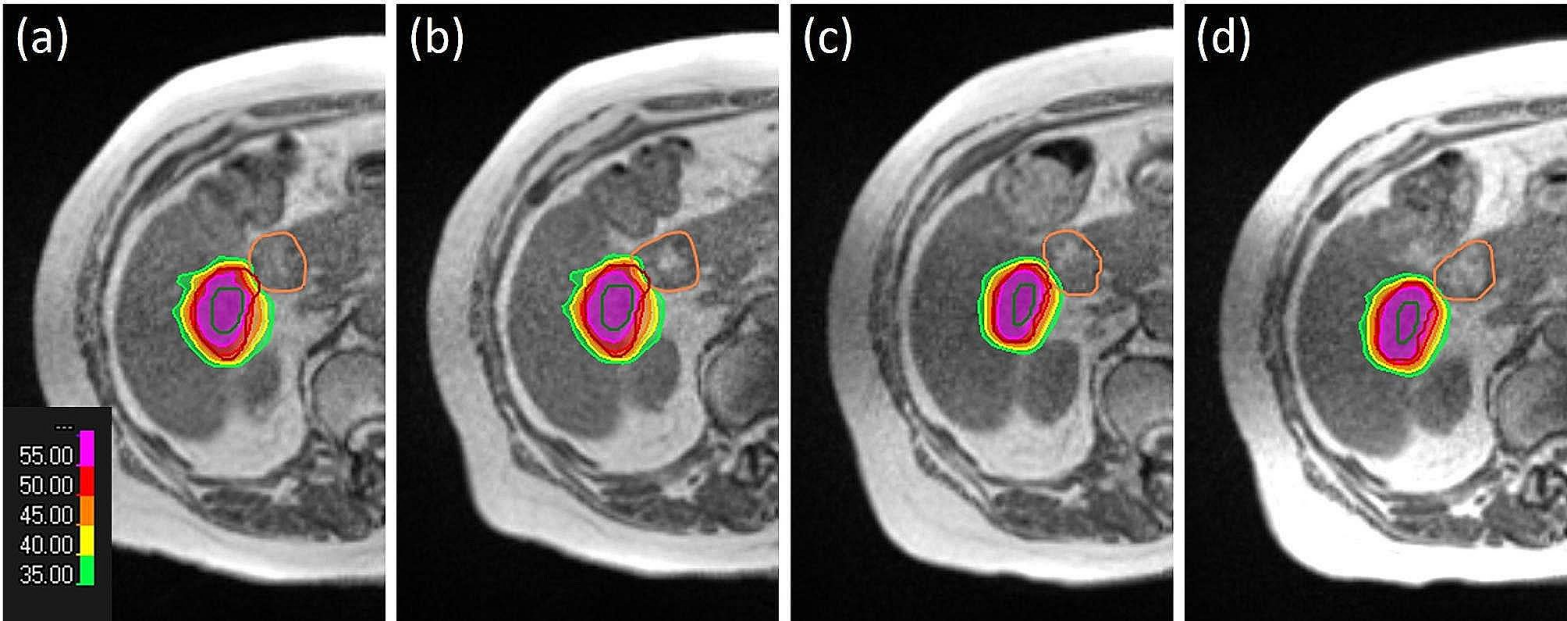
Case example of intrafractional changes to OAR dose. The duodenum dose constraint was met in the adapted dose distribution (**a**), but intrafractional organ movement caused a constraint violation in the pre-irradiation dose distribution (**b**). In another fraction of the same patient, the duodenum constraint was met in both, the adapted (**c**) and the pre-irradiation dose distribution (**d**). The duodenum is depicted in orange, the GTV in green and the PTV in red. The duodenum D_0.5cc_ dose constraint for this prescription is at 35 Gy

A comparison between OAR D_0.5cc_ values in the adapted and pre-irradiation plans in relation to the applied dose constraints for each patient can be found in Fig. 3. The number of OAR dose constraint violations increased significantly between the adapted and the pre-irradiation dose distribution (McNemar’s test, *p* < 0.05). While tolerance dose was only violated in 12/189 cases (6.3%) in the adapted plans, violations occurred in 60/189 (31.7%) cases in the pre-irradiation dose distributions. Furthermore, the pre-irradiation D_0.5cc_ dose value was increased in 62.4% of cases compared to the adapted dose distribution and consequently the respective dose constraint was violated in 50.8% of those cases. A case example can be found in Fig. 4. Overall, a statistically significant increase in the OAR D_0.5cc_ value relative to the respective dose constraint of 4.4% between the adapted plans and pre-irradiation dose distributions was observed using the linear mixed effects model (*p* < 0.001, 95% CI = [2%, 7%]). When evaluated separately, observations of small bowel, duodenum, stomach and esophagus had a higher dose in 67/100, 26/45, 21/39 and 4/5 of analyzed cases. Most violations of dose constraints in the pre-irradiation plan occurred in small bowel with 34 violations and duodenum with 18 violations. Only 5 violations of dose constraints were detected in the stomach and 3 in the esophagus.

### Organs at risk range of movement

**Fig. 5:**
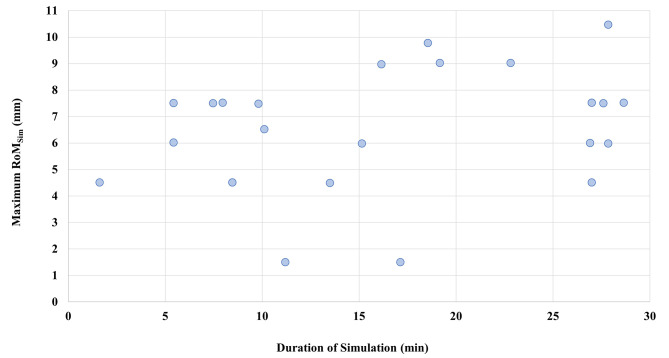
Maximum RoM_Sim_ value over simulation duration for each patient. The duration of evaluated simulation sessions was between 1.6 and 28.7 min and maximum RoM_Sim_ values between 1.5 mm and 10.5 mm were determined. No significant correlation was found between RoM_Sim_ values and the duration of simulation sessions (*p* > 0.05)

RoM_Adapt_ values for all patients varied between 1.5 mm and 22.8 mm with a median RoM value of 6.0 mm (interquartile range: 3.0 mm). Large variations of RoM_Adapt_ were determined for some patients e.g. Patient 4, where RoM_Adapt_ values were between 6.5 mm and 22.8 mm. For other patients RoM_Adapt_ values of all fractions only varied slightly, for example Patient 2 with values between 6.0 mm and 7.5 mm. Maximum RoM_Sim_ values were between 1.5 mm and 10.5 mm. Two patients had low RoM_Sim_ values of only 1.5 mm. In Fig. 5 the maximum RoM_Sim_ values, including all simulation images to determine the value, are plotted over time. Pearson correlation analysis showed no significant correlation between maximum RoM_Sim_ and simulation time (*p* > 0.05). When compared to RoM_Adapt_, RoM_Sim_ was larger or equal in 55.6% of all observations if only the first and last image acquired during simulation were used to determine maximum RoM_Sim_. This could be improved to 67.3% of all observations by using all images acquired during simulation and calculating RoM_Sim_ between each image.

**Fig. 6:**
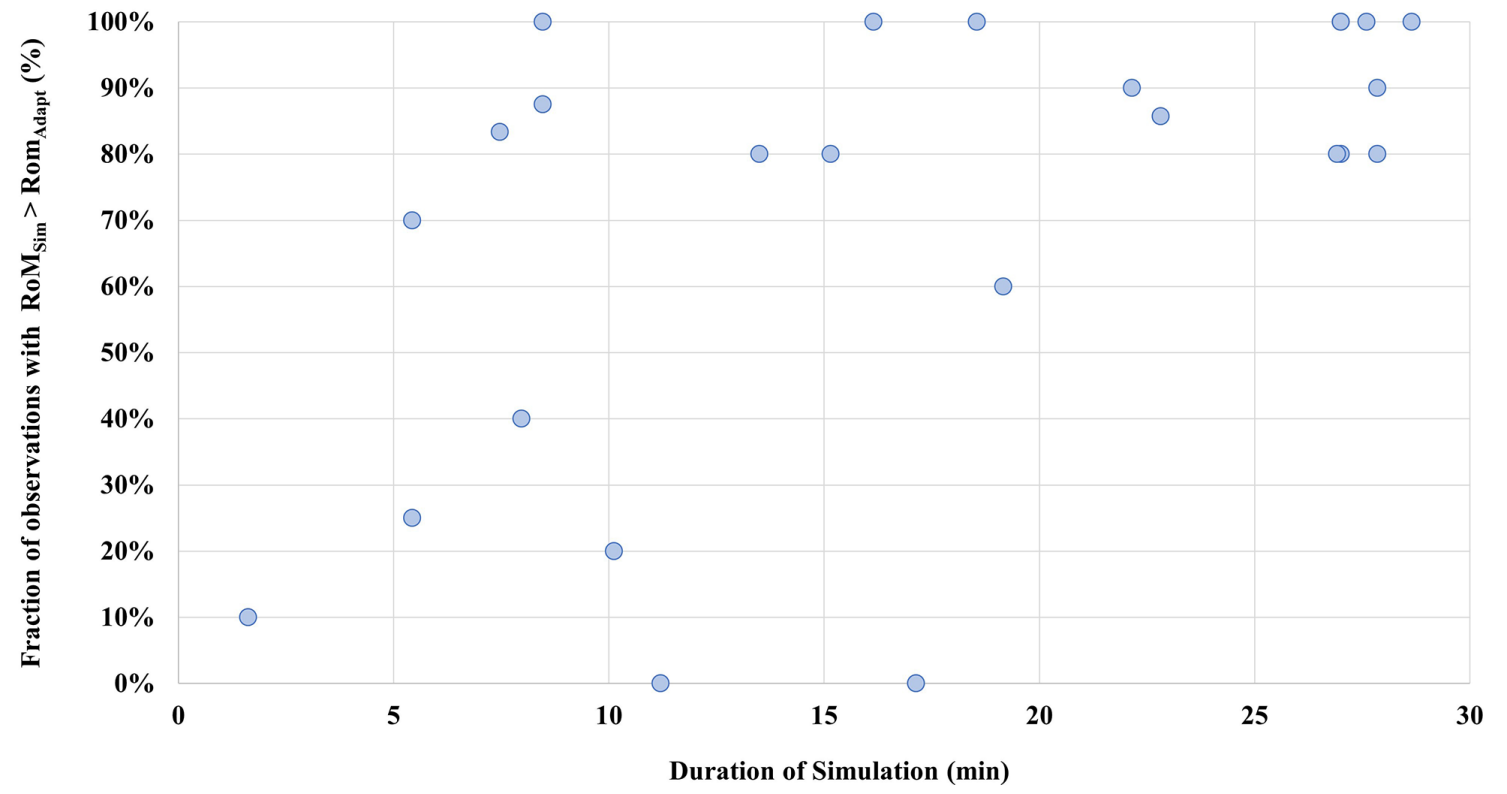
Agreement between RoM_Sim_ and RoM_Adapt_. For each patient, the percentage of fractions where maximum RoM_Sim_ is larger or equal to RoM_Adapt_ is depicted over the duration of respective simulation sessions. Agreement between RoM_Sim_ and RoM_Adapt_ increased significantly for longer simulation sessions (r_P_ = 0.52, *p* < 0.05)

Additionally, the percentage of observations where RoM_Sim_ was equal or larger than RoM_Adapt_ was determined for each patient. A summary of these percentages as well as maximum RoM_Sim_, range of RoM_Adapt_, simulation times and number of simulation images can be found in Table 2. Pearson correlation analysis showed a significant correlation between simulation times and percentage of fractions with RoM_Adapt_ being smaller or equal to RoM_Sim_ (r_P_ = 0.52, *p* < 0.05). A higher duration of simulation sessions led to better agreement between RoM_Sim_ and Rom_Adapt_. A plot of these percentages over simulation times is depicted in Fig. 6. A further analysis on the effect of duration of simulation sessions showed that RoM_Sim_ was larger or equal to RoM_Adapt_ in 89.7% of observations, if simulation times were longer than 17.1 min. In comparison, RoM_Sim_ sufficiently predicted RoM_Adapt_ in only 56.0% of observations for simulation times shorter than 17.1 min. The time of 17.1 min was chosen to split observations, since it represented the mean duration of simulation sessions.

**Table 2:** Overview over treatment simulation data and results of RoM evaluation

Patient No.	Duration Simulation (min)	No. of simulation MR images	Maximum RoM_Sim_ (mm)	Range RoM_Adapt_ (mm)	Median RoM_Adapt_ (mm)	Percentage of observations RoM_Sim_ ≥ RoM_Adapt_
1	18.6	4	9.8	4.9–9.8	6.5	100%
2	27.0	7	7.5	3.0–7.5	7.5	100%
	27.0	7	4.5	3.0–7.5	4.5	80%
	27.0	7	7.5	3.0–6.0	6.0	100%
3	28.7	3	7.5	4.5–7.5	4.5	100%
4	10.1	4	6.5	6.5–22.8	10.6	20%
5	16.2	4	9.0	4.5–9.0	6.0	100%
6	11.2	2	1.5	4.5–10.5	7.5	0%
7	19.2	4	9.0	7.5–13.5	9.0	60%
8	26.9	4	6.0	1.5–13.5	4.5	80%
9	27.6	4	7.5	4.5–7.5	6.0	100%
10	5.4	2	6.0	4.5–13.5	8.3	25%
	5.4	2	7.5	1.5–19.6	5.3	70%
11	7.5	2	7.5	3.0–9.0	6.8	83%
12	15.2	4	6.0	1.5–7.5	3.0	80%
13	8.5	2	4.5	3.0–7.5	3.8	88%
	8.5	2	4.5	3.0–4.5	3.8	100%
14	22.8	4	9.0	3.0–13.5	6.0	86%
15	1.6	2	4.5	4.5–12.0	8.3	10%
16	13.5	2	4.5	3.0–7.5	4.5	80%
17	27.9	6	10.5	3.0–15.0	10.5	80%
	27.9	6	6.0	1.5–9.0	4.5	90%
18	17.1	3	1.5	3.0–10.5	4.5	0%
19	8.0	2	7.5	3.0–10.5	9.0	40%
20	22.2	3	7.5	3.0–9.0	5.3	90%

For each patient duration of simulation, number of images acquired during the simulation session, maximum RoM_Sim_ value and range of RoM_Adapt_ values over all fractions are depicted. Additionally, the percentage of fractions where maximum RoM_Sim_ was larger or equal to RoM_Adapt_ is shown

## Discussion

The comparison between adapted and pre-irradiation OAR doses showed that intrafractional OAR movement in this study strongly impacted dose to OARs directly next to or overlapping with the PTV. A higher dose was found in a majority of analyzed cases (> 60%) in pre-irradiation dose distributions and in about one third of the cases the respective dose constraint of the evaluated OAR was subsequently violated. The observed rate of dose constraint violations for duodenum and small bowel was distinctly higher than for stomach, which might be caused by their larger mobility, especially of the small bowel, compared to the stomach [15, 20].

The proposed margin-based method enabled the determination of the range of OAR movement for a majority of treatment fractions in advance using simulation images. For the evaluated patients, this was especially the case, if simulation took longer than approximately 17 min and multiple MRI images were acquired. Simulation sessions below 17 min were sufficient to get a good estimate of the range of organ movement, but they were less reliable than longer simulation sessions in this analysis.

Two patients showed small organ movement during simulation with a RoM value of only 1.5 mm, while organ movement during adaptation was distinctly larger. For one of these patients, the small bowel moved further away from the PTV during simulation in consecutive MRI_Sim_. However, only OAR movement towards the PTV was considered in this study and as a result these movements were not incorporated in the determination of RoM_Sim_. The other case of small RoM_Sim_ values was probably caused by large differences in stomach filling during simulation and adaptation. While the stomach appeared empty on MRI_Sim_, it was significantly more filled at each treatment fraction and emptied into the duodenum during the adaptation process, thus causing large displacements of the duodenum. Both cases could be prevented in the future by performing patient simulation as well as treatment with an empty stomach, not only in cases where the stomach is directly next to the treated lesion but also for the duodenum as a relevant organ at risk.

Our findings concerning changes to OAR dose and constraint violations are in accordance with those reported by Teoh et al., who observed violation of OAR dose constraints in 37% of post treatment plans [14]. Tyagi et al. observed a slightly higher rate of dose constraint violations in the treatment of locally advanced pancreatic cancer, with a violation rate of 42% for stomach and 52% for small bowel [13]. The higher rate of dose constraint violations could be explained by the use of dose to 0.035 cm³ instead of dose to 0.5 cm³ for evaluation. This smaller volume might be more sensitive to dose changes, particularly in regions with high dose gradients.

However, the actual impact of dose constraint violations on the patient seems to be limited. As previously reported, there were no grade ≥ 3 toxicities observed at our institution in adaptive MR-guided SBRT treatments of adrenal metastases, liver metastases or abdominal lymph nodes [9, 11, 21]. Other studies on treatment of the pancreas [8] as well as other abdominal malignancies [10] did not report higher grade toxicities of gastrointestinal OAR either. One possible explanation could be that the real tolerance dose of OARs is in fact higher than has been assumed so far. Many dose constraints used for MR-guided adaptive treatment are derived retrospectively from observations of SBRT treatments on conventional linacs with no possibility for daily plan adaptation or beam-gating [22–24]. Prospective studies are needed in order to determine the full potential of safe dose escalation in adaptive SBRT treatments. This could also lead to improved PTV coverage in those cases where OARs are overlapping with the PTV, because often the coverage needs to be reduced in order to meet OAR dose constraints.

Although intrafractional OAR movement does not seem as relevant for current dose prescription concepts due to low OAR toxicity, they could become more relevant when dose to the tumor is further escalated. A dose escalation study for locally advanced pancreatic cancer is currently recruiting, prescribing 50 Gy in five fractions to the pancreas [25]. Furthermore, the phase II RASTAF study examines dose escalation in MR-guided treatment of primary or secondary liver metastases, although higher doses up to 60 Gy in 6 fractions remain limited to metastases far from OARs for now [26].

The method used to determine range of movement in this study could be directly translated into the generation of isotropic OAR margins, which could then be applied for plan optimization. However, one major limitation of isotropic margins for mobile OARs like small bowel is that the expanded OAR often encompasses a large area, even though the OAR actually might not occupy many parts of this area most of the time. In cases where the OAR is overlapping with the PTV this could lead to heavily impaired PTV coverage. Many approaches to generate more individualized margins like probability based PRVs [15, 16] are quite complex and not easily applicable in a clinical routine, though. Further research is needed to assess how more complex organ movement, determined by the acquisition of multiple simulation images, can be predicted and accounted for in adaptive treatments, especially in the context of dose escalation trials. Furthermore, in this study, patients were selected retrospectively based on the position of OARs in relation to the PTV and therefore the number of simulation images available for each patient varied strongly. To verify our results, further studies are warranted with defined number of simulation images and time.

The evaluated simulation data suggests that compensation for intrafractional organ movement might be necessary even for short treatment times. Large movement of mobile organs at risk like small bowel can occur anytime during patient treatment and is not limited to long adaptation times. This is supported by data from some patients where large variations of OAR position between 4.5 mm and 7.5 mm could be observed after only six minutes. In a previous study by Liu et al. gastrointestinal organ movement of up to 2 mm per minute [15] was reported, which could lead to large OAR displacement in a short time. In addition, Uchinami et al. determined margins with median values of up to 14 mm to account for gastrointestinal movement using CT images acquired within a median timeframe of 12.3 min [20]. Since treatment delivery of abdominal lesions in MRgRT is routinely performed in inspiration breath-hold with automatic beam gating, beam-on times alone can already be within this timeframe. Previous studies reported median beam-on times of 10.5 min or more, without taking into account the duration of the adaptation process [10, 13, 27, 28]. Therefore, intrafraction movement of gastrointestinal OARs might still need to be considered and also compensated for, even if the duration of adaptation can be significantly reduced in the future.

## Conclusions

In MR-guided adaptive radiotherapy of abdominal lesions intrafractional movement of OARs overlapping with the PTV is affecting OAR dose significantly and leads to violation of dose constraints in some cases. MR images acquired during treatment simulation can be used to predict the range of OAR movement during adaptation in advance and could also be used to generate PRVs for individualized treatment planning. Evaluation of organ movement on simulation images also indicates that intrafractional organ movement might still be relevant, even if adaptation times can be considerably shortened in the near future.

## Data Availability

No datasets were generated or analysed during the current study.
